# Addressing Thalassaemia Management from Patients’ Perspectives: An International Collaborative Assessment

**DOI:** 10.3390/medicina60040650

**Published:** 2024-04-18

**Authors:** Eleftheria C. Economidou, Michael Angastiniotis, Demetris Avraam, Elpidoforos S. Soteriades, Androulla Eleftheriou

**Affiliations:** 1 Department of Pediatrics, Larnaca General Hospital, 6043 Larnaca, Cyprus; eleftheria.economidou@gmail.com; 2 Thalassaemia International Federation (TIF), 2007 Nicosia, Cyprus; michael.angastiniotis@thalassaemia.org.cy (M.A.); thalassaemia@cytanet.com.cy (A.E.); 3 Department of Public Health, Policy and Systems, University of Liverpool, Liverpool L69 3BX, UK; demetris.avraam@liverpool.ac.uk; 4 Healthcare Management Program, School of Economics and Management, Open University of Cyprus, 33 Giannou Kranidioti Ave., 2220 Nicosia, Cyprus; 5 Department of Environmental Health, Environmental and Occupational Medicine and Epidemiology (EOME), Harvard T.H. Chan School of Public Health, Boston, MA 02115, USA

**Keywords:** epidemiology, thalassaemia, questionnaire, health services, ITHACA, international survey

## Abstract

*Background and Objectives*: The effective management of chronic diseases, particularly hereditary and rare diseases and thalassaemia, is an important indicator of the quality of healthcare systems. We aimed to assess healthcare services in different countries for thalassaemia patients by using publicly available health indicators and by surveying thalassaemia patients and their caregivers. *Materials and Methods*: We reviewed official worldwide databases from the WHO, World Bank, and scientific resources, and we used a structured patient-tailored self-completed questionnaire to survey thalassaemia patients and their caregivers in 2023. *Results*: A total of 2082 participants were surveyed (mean age, 27 years; males, 42%). About 1 in 4 respondents did not complete high-school education, while 24% had a bachelor’s degree. About a third of respondents were married and were in either full- or part-time employment. The vast majority (~80%) had initiated transfusion therapy between 1 and 4 years of age. Only 42% reported no delays in receiving blood transfusion, while 47% reported occasional delays and 8% serious delays. About half of patients reported being very satisfied (11%) or satisfied (38%) with the quality of services provided, while 1 in 3 patients reported being unsatisfied or very unsatisfied, and that their access to treatment was difficult or very difficult due to traveling expenses and the high cost of treatment. *Conclusions*: Important improvements in the care of thalassaemia patients have been documented during the past few decades. Nevertheless, additional focus is required through national healthcare systems to effectively address the many unmet needs revealed by our recent survey, as well as to achieve satisfactory patient outcomes.

## 1. Introduction

Thalassaemia syndromes are a major burden to healthcare systems, especially where they are prevalent in terms of patient numbers. Assessment of the burden and the response of health systems to these lifelong chronic conditions remains an important challenge, particularly since most countries with high disease prevalence have limited resources, which means that they can provide limited services for these demanding conditions. Basic needs include regular blood transfusions and daily medications for iron chelation, as well as complex clinical monitoring. For patients to survive and thrive in any country and receive equitable services, it is necessary to take into consideration thalassaemia epidemiology and the variations in patient numbers due to new births, deaths, and migrations. It is also necessary to understand the health priorities in each country and how resources are distributed for clinical and public health needs.

Various indices are used for such assessments. However, there is still much global debate on the appropriate approach to weigh the various individual indicators of health. Summary measures such as years of potential life lost, disability-adjusted life years (DALYs), or disability-adjusted life expectancies (DALEs) may be reasonable approximations of individual indicators, although their limitations have been recognised [1].

Organisations such as the Thalassaemia International Federation are advocates for health service quality and continuous improvement. Therefore, knowledge of indicators and health systems is required to make advocacy effective. In this context, as a state that encompasses physical, behavioural, spiritual, and socioeconomic well-being based on the philosophy of individual-, family-, and community-centredness within a broader framework of healthcare systems, whole patient health constitutes an important outcome [2]. In parallel, public health encompasses all evidence-based efforts employed by society to protect and promote health and well-being, prevent disease and disability, prolong life expectancy, and enhance quality of life for the population as a whole [3,4]. Globally, thalassaemia patients are not equally treated according to these principles, yet within the realm of healthcare services, chronic diseases deserve particular attention since they have a significant impact on the health of individuals and populations alike, requiring the devotion of extensive health and societal resources [5,6,7,8].

Thalassaemia represents an important chronic–genetic disorder, the effective management of which reflects, to a great extent, the quality of healthcare services provided to the population from different perspectives, namely prevention, control, and management [9,10,11,12]. Pioneer countries, such as Italy, Greece, and Cyprus, in providing ideal services to thalassaemia patients, have developed disease-specific national programmes that promote, amongst other things, the development of reference centres that allow health professionals to come together to provide the very best treatment and care for thalassaemia patients. This support is often resourced exclusively by the national healthcare systems, safeguarding patients from the financial burden of such complicated and expensive care. Considering that many thalassaemia patients around the world are managed in peripheral/rural centres with limited resources, the need for expert networking and tele-consultations has become imperative [13].

Appropriate healthcare indicators constitute reliable tools that provide timely information, particularly on the health status of the population as a whole and its particular segments, and can be used to establish public health priorities, mobilise funds, monitor the performance of particular actions and programs, and inform related policy decision making [3,14]. However, these indicators frequently ignore the patient’s voice, which can be assessed through patient-centred surveys that provide valuable insight into the gaps and limitations of the services provided to those in need [15,16]. Several surveys on particular health aspects of thalassaemia patients from different countries have been published in the international literature [17,18,19]. However, there is scarce information on a universal global assessment of such patients.

Our international collaborative assessment aimed to summarise a number of officially selected individual and population health indicators from several different countries to assess the quality and quantity of healthcare services provided to thalassaemia patients. Furthermore, in the context of this study, we also aimed to evaluate healthcare services for thalassaemia management from patients’ and caregivers’ experience through a survey, using a tailored designed and self-completed questionnaire.

## 2. Materials and Methods

The project was implemented in 2023 as commissioned by the Thalassaemia International Federation (TIF) to the Open University of Cyprus, School of Economics and Management, Healthcare Management Program. It comprised two complementary parts. The first part included a comprehensive international healthcare management report, finalised through a detailed review of official worldwide databases from different international organisations and scientific resources using readily available health indicators. The second part included the design, development, and implementation of a survey to assess the quality of Thalassaemia patient management from a clients’ perspective through an internationally self-completed questionnaire distributed in 2023.

### 2.1. The International Healthcare Management Report

This report aimed to collect a number of healthcare indicators regarding the different healthcare services provided to the general population and thalassaemia patients in particular from several different countries around the world. A list of countries included in the first international report is presented in Section S1. Data regarding public health indicators from countries distributed in five continents were collected from official international sources such as the World Health Organization (WHO) [20] and the World Bank [21], as well as PubMed, and the Web of Science. The following parameters and public health indicators were considered: population; gross domestic product (GDP); World Bank income rank; expected number of thalassaemia births per 1000 live births; universal health coverage; chronic diseases/non-communicable diseases; crude birth rates; current health expenditure (% of GDP); government expenditure on education (% of GDP); poverty; life expectancy at birth; infant mortality rate; crude death rate; cause of death by communicable diseases and maternal, prenatal, and nutrition conditions; healthy life years; diabetes prevalence; prevalence of tobacco use; prevalence of obesity; alcohol consumption; and per-capita coverage of social insurance programs.

### 2.2. The International Thalassaemia Collaborative Assessment Patient Survey

The survey involved the design and development of a data collection instrument (a structured anonymous questionnaire) that evolved following repeated rounds of review by faculty at the Open University of Cyprus, Healthcare Management Program, along with a research fellow hired to work on the project, as well as the medical consultant of the Thalassaemia International Federation and its research officers.

#### 2.2.1. Data Collection Instrument

The questionnaire used in the survey is presented in Section S2 in English. The international assessment was facilitated by a survey questionnaire that was designed as a tailored, flexible instrument targeting thalassaemia patients and/or their caregivers. The development phase of the questionnaire included a collaborative design process. A team from both the Open University of Cyprus and the Thalassaemia International Federation worked cooperatively to develop the different sections of the survey tool, which was approved by the TIF Executive Director, who spearheaded this creative iterative process. The questionnaire comprised three different sections. The first included demographic and other characteristics of thalassaemia patients, while the second focused on different medical parameters. The third and final extensive section included several questions on the quality and quantity of the services received by thalassaemia patients along with information on the financial aspects of services and patient satisfaction.

#### 2.2.2. Survey Translation, Informed Consent, and Deployment

To ensure global inclusivity, the survey was then meticulously translated into twenty-five (25) different languages, ranging from Albanian to Visayas, including Arabic, Chinese, Farsi (Persian), French, German, Greek, Hindi, Indonesian, Italian, Khmer, Lao, Malay, Nepali, Sinhala, Spanish, Tagalog, Turkish, Urdu, and Vietnamese. A list of countries along with their survey language used is presented in Section S3. Prior to participant engagement, a transparent and comprehensive informed consent disclaimer was presented, which clearly outlined the eligibility criteria for respondents, emphasising the intended audience of individuals with thalassaemia aged 15 or older, or parents/caregivers of patients under 15 years of age. The distribution of the thalassaemia survey was implemented via online and paper-based approaches to many different countries around the world. Survey Monkey was chosen as the survey deployment platform due to its accessibility and user-friendly interface. The project was implemented based on a written agreement and approval by the Thalassaemia International Federation Executive Office and the Dean’s office of the Open University of Cyprus.

The survey’s purpose was to assess healthcare services provided around the world from patient and caregiver perspectives to contribute to the TIF’s mission of advocating for universal and comprehensive management services for all. The project’s goals were communicated to prospective survey participants who were also assured of the confidentiality and anonymity of their provided information, fostering trust and openness in their responses. The TIF strategically leveraged its extensive network and longstanding presence in the field of global thalassaemia to facilitate the distribution of the survey at the national level. This approach was grounded in experiences gained from previous surveys conducted in 2014, 2017, and 2022. Therefore, we emphasised personal contact with peers as a more effective means of soliciting responses than traditional email outreach. Delegates were carefully trained to employ the right approach in collecting data from eligible individuals. This tailored strategy was implemented for each language and country, fostering a nuanced and culturally sensitive approach to survey engagement.

#### 2.2.3. Data Management—Statistical Analyses

A strategic decision aimed to maximise respondent participation by offering an intuitive and straightforward experience. The Survey Monkey platform’s versatility allowed for the survey to be available in various languages, promoting inclusivity across linguistic and cultural backgrounds. The data collected were downloaded into an electronic database that was used for statistical analyses.

The survey was conducted between June and September 2023 and utilised specialised software to ensure secure and high-quality data collection. Where applicable, local TIF contacts facilitated the transfer of responses from hard copies to the online data collection tool, with a notable collection of 300 hard copies from Bangladesh sent to the TIF electronically. Hard-copy responses were then integrated into the online Survey Monkey platform. A TIF-skilled officer was entrusted with transferring paper-based responses to maintain accuracy, compatibility, and fidelity. The data collected from hard copies were downloaded in a codified format and seamlessly integrated with the online responses, ensuring consistency and uniformity of the overall project dataset.

Post-collection data were downloaded from Survey Monkey in both full-text and codified formats. A research associate of the Open University of Cyprus and collaborator of the TIF was responsible for data management and statistical analyses. The goal was to transform the opinions and experiences of patients and families into tangible insights and interpretable results for public and policymaker consumption, thereby informing the TIF’s strategic positioning and policymaking. Statistical analyses were performed with the “R” open-source statistical software.

## 3. Results

In summarising the healthcare management report, we used publicly available, official, web-based databases on different population health indicators for the general population and thalassaemia patients in particular. To assess the quality and quantity of healthcare services provided by different countries around the world, we tabulated a total of 48 different health indicators.

In Table 1, we present the basic public health and healthcare indicators collected that exert a significant impact and/or reflect on the health services provided by each country. We present collected information on country population, infant mortality, life expectancy, country gross domestic product (GDP), and government expenditure per capita. In Table 2, we further tabulate additional public health indicators including government expenditure on education and health. In addition, we present the World Bank income rank for each country along with the expected number of thalassaemia births per 1000 live births.

We evaluated a total of 48 countries from all continents. The population in millions in the survey countries ranged from 0.5 (Maldives) to 1417 (India), while the documented infant mortality rate per 1000 live births ranged from 2 (Singapore) to 53 (Pakistan). Life expectancy ranged from 64 (Yemen) to 84 years of age (Singapore, Italy, Spain, and Switzerland). The current health expenditure in different countries as a percentage of the gross domestic product ranged from 2.4% (Brunei) to 18.8% (USA). A total of 42% of countries were ranked by the World Bank as high-income, 27% were ranked as upper/medium-income, and 31% were ranked as low/medium-income. Finally, the expected number of thalassaemia births per 1000 live births ranged from 0.001 (Honduras) to 8.9 (Maldives).

**Figure 1:**
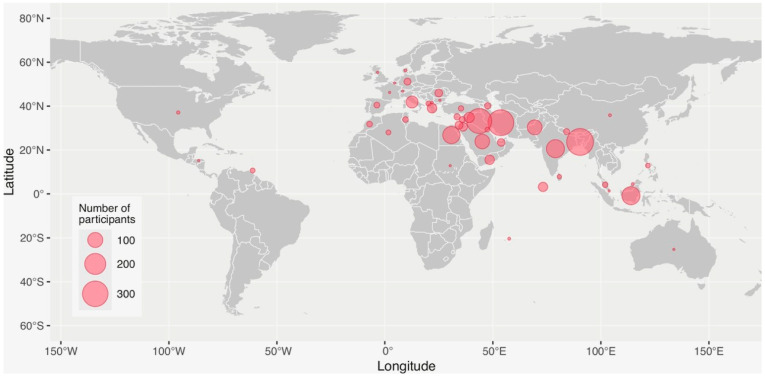
Geographical distribution of participants in the TIF’s international thalassaemia collaborative assessment survey, created using the ggplot2 package in “R”. The circle radius represents the number of survey participants from each country.

In the context of the international collaborative assessment survey, we received responses from a total of 2082 participants, both thalassaemia patients and caregivers who participated in the global survey, 500 of whom were from European Union countries. We included information from 48 countries around the globe. In Figure 1, we present the geographical distribution of survey participants in our international survey from different continents, based on the number of participants from each country.

In Table 3, we present a descriptive distribution of demographic and other characteristics of the participants as derived from the survey, as well as the frequencies of their answers to questions in relation to medical information and the quality of services provided to thalassaemia patients. The mean age of respondents was 27 years of age. About 70% were patient respondents and the remainder were caregivers, and 42% were males. About 1 in 4 respondents did not complete high-school education and another 24% had a bachelor’s degree. A third of respondents were married and a similar percentage were in either full- or part-time employment. The vast majority of respondents (~80%) had initiated transfusion therapy at 1–4 years of age and about half of them (47%) were transfused when their haemoglobin levels were between 8 and 9 mg/dL. Only about 42% reported that they had no delays in receiving blood transfusions on time while 47% reported occasional delays and 8% reported transfusion delays that could put them at risk of severe anaemia.

In Table 3, we also present information on clinical parameters of thalassaemia patient management. Blood filtration was performed at the bedside for one in four patients, while chelation therapy was initiated at age 5 or older for one in three thalassaemia patients. Chelation therapy was reported as being given regularly to 71% of the international sample of thalassaemia patients, while 8.5% had never received chelation therapy. A similar percentage (71%) reported that their ferritin level was examined every 3 to 4 months, while 13% reported that their ferritin level was checked either every year or never. Additional information is also provided about the ferritin levels themselves, cardiac iron measurements by T2*, and liver iron concentrations.

Overall, about half of the patients reported being very satisfied (11%) or satisfied (38%) with the quality of provided services, while one in three patients reported being unsatisfied or very unsatisfied with the quality of medical services. In parallel, one in three patients reported that their access to treatment was difficult or very difficult due to the high cost of the treatment itself and the high cost of traveling to medical centres for treatment. Finally, about half of the patients (>55%) reported that they were losing more than 10 days per year from school or work to attend treatment sessions.

Finally, in Table 4, we present a number of selected survey parameters listed in the previous table in cumulative forms, as tabulated by the WHO regions. It is evident that there are stark differences between the different WHO regions in all parameters examined. As documented by the survey findings, the mean age distribution, marital and employment status, educational attainment, and access to health services and treatment differ significantly between the different regions. Regions with better healthcare services, such as Europe, show a higher mean age of patients; higher educational, marital, and employment status; and better access to treatment that is provided by state healthcare services.

## 4. Discussion

To our knowledge, this is one of the first international surveys of thalassaemia patients and their caregivers, conducted in 2023, aiming to capture the extent as well as the quality and quantity of healthcare services provided to this specific patient population in various countries around the world. In the current article, we describe the basic findings of this international assessment as well as the results of the extensive survey.

Overall, the majority of thalassaemia patients around the world are receiving good-quality care including timely blood transfusions [22], chelation therapy [23], adequate disease-specific medical monitoring, and appropriate imaging, and about half of them report being satisfied or very satisfied with the medical management provided [24]. Nevertheless, based on our findings, a sizeable percentage of the study participants lack comprehensive services, face difficulties in accessing guideline-based recommended care, face barriers in receiving timely care due to financial difficulties, and are not satisfied with the level of healthcare services they receive.

The Thalassaemia International Federation (TIF), as an international federation of 200 national patient support organisations from over 60 countries around the world, is strategically involved in supporting the development and implementation of national prevention and control programmes, and promoting optimal management for patients with haemoglobin (Hb) disorders. The TIF’s goal focuses on securing equal access to good-quality healthcare services for all patients with haemoglobin disorders around the world [25]. The current research, commissioned by the TIF in collaboration with the Open University of Cyprus, is an attempt to globally map the status of thalassaemia patients with respect to their personal and social characteristics, as well as the quality and quantity of clinical management and associated healthcare services provided by different countries around the world.

The TIF collaborates with different agencies and organisations of the European Union, WHO headquarters, and regional offices, promoting public health and disease-specific policies for rare and hereditary blood disorders in general and thalassaemia in particular. Furthermore, TIF works closely with clinical specialists in different medical disciplines to enhance the quality of medical services provided to thalassaemia patients around the world. In addition, TIF is actively cooperating with universities and academic centres to support and promote scientific research, associated conferences, and scientific publications to advance knowledge in the field of thalassaemia prevention, control, and clinical management. Specifically, TIF medical advisors and international experts have updated the third publication on the clinical management of both α and β thalassaemia syndromes [26], which has set a comprehensive quality standard that should be followed to achieve the best possible outcomes among thalassaemia patients worldwide. Furthermore, the organisation is also active in Europe, supporting the inclusion and promotion of Hb disorders in the communities of every EU country member which, until recent years, were either considered rare and hence addressed in the context of rare disease recommendations, or were concentrated amongst the migrant population and hence their needs were left unaddressed. EU-funded projects (the third Health and the EU4Health Program—Thalia project) have greatly supported TIF in advocating and recommending policies for effectively addressing this hereditary disorder [27]. Finally, a highlighted focus of TIF activities relates to the support of patients’ rights to cross-border healthcare in Europe and the promotion of the establishment of reference centres and networking across the globe.

Based on the findings of our study, we conclude that several past improvements in the management of thalassaemia patients are benefitting many patients around the world, even in low-income countries. At the same time, a sizeable percentage of thalassaemia patients are experiencing difficulties in receiving standard treatment modalities as well as the laboratory and imaging examinations required to monitor their overall health, treatment, and potential complications. Our survey reveals areas that require additional focus from the clinical and public health community as well as from policymakers around the world. One of the important findings of the first phase of our study is the significant discrepancies seen in the infant mortality rates between different countries and particular continents. For example, the highest infant mortality rates are seen in some African countries. Similar negative findings are also documented for thalassaemia patients in these countries. Thalassaemia patients’ views and satisfaction or anxiety concerning the services offered are rarely understood or taken into consideration. This survey is an initial analysis of global patient opinions on these important aspects of their medical and overall care.

The project’s strength is related to the presentation of thalassaemia patient perspectives with respect to the services received in different countries around the world. Due to the difficulties in globally accessing thalassaemia patients, we used a convenient sample process. Nevertheless, the large number of countries included in our survey and the diversity of country settings used in collecting patient and caregiver feedback provide a fair representation of the overall thalassaemia patient population and their health-related experiences. In addition, although some countries had very few participants, the cumulative sample findings provide an informative picture of patient perspectives. Hence, the large amount of information collected in the survey does not allow us to analyse the results in detail in the present report.

Challenges in addressing timely and good-quality treatment (as recommended by international experts and TIF guidelines), particularly including access to adequate blood, delays in receiving blood transfusion therapy, concerns about the quality and safety of blood, methods of appropriately processing blood, access to chelation therapy and accurate and reliable monitoring of iron load, access to specialised centres and healthcare professionals with expertise in the field, and stigmatisation and its impact on the professional and personal lives of thalassaemia patients, are a few of the reported difficulties faced by patients and their caregivers in many different countries. Although knowledge and expertise are abundant in the Western world, the rarity of the disease creates many challenges in the establishment of national disease-specific strategies and policies. The recent intense migration flow has intensified such gaps. Overall, considerable efforts on behalf of the WHO, regional and national authorities, healthcare professionals, and patients and their families are required to improve existing and develop new disease-specific policies for effectively addressing the above shortcomings.

The limitations of our study include the collection of information from different countries, leading to concerns regarding the reliability of the information collected due to the different sample selection processes used in each country. Consequently, study participants were underrepresented in some countries. The information collected originated from two separate population groups, namely patients themselves and caregivers. The survey questionnaire was developed ad hoc for this study, and the validity was not examined. In addition, there were some missing values regarding the different parameters included in our survey.

## 5. Conclusions

Despite demonstrating improvements in many areas of social parameters and the medical management of thalassaemia patients compared to previous reports, the findings of the current survey document significantly fewer benefits, primarily in the area of prevention. Furthermore, stark discrepancies exist between different world regions with respect to the quality and quantity of disease-specific healthcare services provided to thalassaemia patients that threaten the health, quality of life, and social integration of patients with this condition. These observations are mainly seen in, but not confined to, the developing areas of the world, as reported by patients themselves.

## Figures and Tables

**Table 1:** Basic health indicators of different countries in the survey.

A/A	Country	Population (Millions)	Infant Mortality Rate (per 1000 Live Births)	Life Expectancy	GDP (Current USD) (Billions)	GDP per Capita (Current USD)
Europe						
1.	Albania	2.8	8.41	78	18.9	6803
2.	Belgium	11.3	3.0	82.46	579	51,166
3.	Bulgaria	6.7	5	72.8	89	13,773
4.	Cyprus	1.26	2	82	28.4	31,284
5.	Denmark	5.9	3	82	395	68,037
6.	France	64.7	3	83.35	278.3	40,964
7.	Germany	83.3	3	82.18	407.2	51,073
8.	Greece	10.3	3	82	219	20,732
9.	Italy	58.8	2	84.2	201	34,158
10.	Romania	19.9	5	75.14	301	15,892
11.	North Macedonia	2.1	4.65	75	13.6	6591
12.	Spain	47.5	3	84.0	139.8	29,350
13.	Switzerland	8.8	3	84.4	808	93,525
14.	Turkey	85.8	8	78.7	906	10,616
15.	United Kingdom	67.7	4	82.3	307	45,850
Middle East						
16.	Yemen	34.4	47	64.5	na *	702
17.	Jordan	11.3	12.56	75	47.5	4204
18.	Kuwait	4.3	7.48	79	184.6	43,233
19.	Saudi Arabia	36.4	5.75	78	1108.1	30,436
20.	Lebanon	5.5	7.05	76	23.1	4136
21.	Syria	23.2	na	76.3	na	537
22.	Palestine (West Bank and Gaza)	5.0	13 (World Bank 2021)	74.3	19.1	3789
23.	United Arab Emirates	9.4	5.45	80.4	507.5	53,757
Africa						
24.	Algeria	44.9	19.16	77.3	191.9	4273
25.	Egypt	111.0	16.23	70	476.7	4295
26.	Mauritius	1.3	15.31	75.7	12.9	10,216
27.	Morocco	37.5	15.42	74	134.2	3527
28.	Tunisia	12.4	14.03	76.9	46.7	3776
29.	Sudan	48.1	39	66.1	51.66	
Asia						
30.	Azerbaijan	10.2	16.61	73.6	78.7	7736
31.	Bangladesh	171.2	22.91	73.9	460.2	2688
32.	Brunei Darussalam	0.45	10	74.5	16.7	37,153
33.	China	1412.2	5.05	78	17,963.2	12,720
34.	India	1417.2	25.49	67	3385.1	2388
35.	Indonesia	275.5	18.88	68	1319.1	4788
36.	Iran	88.6	10.87	76.9	388.5	4387
37.	Iraq	44.5	20.75	70	264.2	5937
38.	Malaysia	33.9	6.46	75	406.3	11,971
39.	Maldives	0.52	5.10	81	6.2	11,817
40.	Nepal	30.5	22.82	68	40.8	1336
41.	Pakistan	235.8	52.78	66	376.5	1596
42.	Philippines	115.6	20.47	69	404.3	3498
43.	Sri Lanka	22.2	5.77	76	74.4	3354
44.	Singapore	6.0	2	84.27	467	82,808
America						
45.	Honduras	10.6	14	73.7	31.7	3040
46.	USA	338.3	5	79.7	254.63	76,399
47.	Trinidad &Tobago	1.5	14.60	73	27.9	18,222
48.	Australia	26.4	3	83.73	362	64,491

* Not available.

**Table 2:** Public health indicators of different countries in the survey.

A/A	Country	Government Expenditure on Education, Total (% of GDP)	Current Health Expenditure (% of GDP)	World Bank Income Rank	Expected Thal. Births/1000 Live Births (Est. from Carrier Rate)
Europe					
1.	Albania	3.1	6.66	Upper/mid income	0.625/1000
2.	Belgium	6.2	11.6	High income	0.002/1000
3.	Bulgaria	4.5	8.52	High income	0.152/1000
4.	Cyprus	5.6	8.1	High income	4.9/1000
5.	Denmark	6	10.58	High income	0
6.	France	5.2	12.21	High income	0.0016/1000
7.	Germany	4.5	12.81	High income	0.0017/1000
8.	Greece	4.1	9.51	High income	1.64/1000
9.	Italy	4.1	9.45	High income	0.462/1000
10.	Romania	3.3	6.27	High income	0.025/1000
11.	North Macedonia	3.3	7.89	Upper/mid income	0.169/1000
12.	Spain	4.6	10.71	High income	0.067/1000
13.	Switzerland	5.0	11.8	High income	0.004/1000
14.	Turkey	2.8	4.62	Upper/mid income	0.121/1000
15.	United Kingdom	5.3	11.94	High income	0.0018/1000
Middle East					
16.	Yemen	na *	4.25	Low/mid income	0.484/1000
17.	Jordan	3.2	7.47	Upper/mid income	0.306/1000
18.	Kuwait	6.6	6.31	High income	0.121/1000
19.	Saudi Arabia	7.8	5.54	High income	0.171/1000
20.	Lebanon	1.7	7.95	Upper/mid income	0.132/1000
21.	Syria	na	3.0	Low/mid income	0.625
22.	Palestine (West Bank and Gaza)	5.3	-	Low/mid income	0.4/1000
23.	United Arab Emirates	3.9	5.67	High income	0.23/1000
Africa					
24.	Algeria	7.0	6.32	Low/mid income	0.1/1000
25.	Egypt	2.5	4.36	Low/mid income	0.7/1000
26.	Mauritius	4.9	6.66	Upper/mid income	0.37/1000
27.	Morocco	6.8	5.99	Low/mid income	0.07/1000
28.	Tunisia	7.3	6.34	Low/mid income	0.122/1000
29.	Sudan	1.0	3.0	Low/mid income	0.38/1000
Asia					
30.	Azerbaijan	4.3	4.61	Upper/mid income	0.344/1000
31.	Bangladesh	2.1	2.63	Low/mid income	2.1/1000
32.	Brunei Darussalam	4.4	2.39	High income	0.47/1000
33.	China	3.6	5.59	Upper/mid income	0.283/1000 (South only)
34.	India	4.5	2.96	Low/mid income	0.58/1000
35.	Indonesia	3.5	3.41	Low/mid income	2.13/1000
36.	Iran	3.6	5.34	Upper/mid income	0.576/100
37.	Iraq	4.7	5.08	Upper/mid income	0.576/1000
38.	Malaysia	3.9	4.12	Upper/mid income	0.377/1000
39.	Maldives	5.8	11.35	Upper/mid income	8.9/1000
40.	Nepal	4.2	5.17	Low/mid income	1.28/1000
41.	Pakistan	2.4	2.95	Low/mid income	0.99/1000
42.	Philippines	3.7	5.61	Low/mid income	0.024/1000
43.	Sri Lanka	1.9	4.07	Low/mid income	0.18/1000
44.	Singapore	2.4	6.0	High income	0.2/1000
America					
45.	Honduras	5.4	9.1	Low/mid income	0.001/1000
46.	USA	5.4	18.82	High income	0.004/1000
47.	Trinidad &Tobago	4.1	7.31	High income	0.306/1000
48.	Australia	5.6	10.65	High income	0.012/1000

* Not available.

**Table 3:** Information on demographics, medical information, and quality of services derived from TIF’s international survey of thalassaemia patients and their caregivers.

Study population	
*n*	2082
Age (in years)	
Mean, (SD)	26.9 (12.6)
Missing (*n*, %)	275 (13.3%)
Patient/Parent	
Patient (*n*, %)	1465 (70.7%)
Parent (*n*, %)	582 (28.1%)
Missing (*n*, %)	24 (1.2%)
What is your gender?	
Male (*n*, %)	877 (42.3%)
Female (*n*, %)	1087 (52.5%)
Missing (*n*, %)	107 (5.2%)
Which of the following categories best describes your employment status?	
Employed, working full-time (*n*, %)	489 (23.6%)
Employed, working part-time (*n*, %)	188 (9.1%)
Not employed, looking for work (*n*, %)	528 (25.5%)
Not employed, not looking for work (*n*, %)	517 (25.0%)
Retired (*n*, %)	69 (3.3%)
Disabled, not able to work (*n*, %)	232 (11.2%)
Missing (*n*, %)	48 (2.3%)
Which of the following best describes your current relationship status?	
Married (*n*, %)	705 (34.0%)
Widowed (*n*, %)	28 (1.4%)
Divorced (*n*, %)	23 (1.1%)
Separated (*n*, %)	24 (1.2%)
Cohabiting (*n*, %)	664 (32.1%)
Single, never married (*n*, %)	532 (25.7%)
Prefer not to answer (*n*, %)	53 (2.6%)
Missing (*n*, %)	42 (2.0%)
What is the highest level of school you have completed or the highest degree you have received?	
Less than high school degree	478 (23.1%)
High school degree or equivalent (e.g., GED)	456 (22.0%)
Some college but no degree	161 (7.8%)
Bachelor’s degree	497 (24.0%)
Master’s degree	158 (7.6%)
Doctoral degree	21 (1.0%)
Trade school	35 (1.7%)
Missing (*n*, %)	265 (12.8%)
If you are a patient, what is your diagnosis? If you are a parent, what is the diagnosis of your child?	
Other (*n*, %)	274 (13.2%)
Beta thalassaemia major (*n*, %)	1510 (72.9%)
Beta thalassaemia intermedia (*n*, %)	250 (12.1%)
HbH disease (*n*, %)	11 (0.5%)
Missing (*n*, %)	26 (1.3%)
At what age did you start transfusion therapy?	
1–4 years old (*n*, %)	1647 (79.5%)
5–6 years old (*n*, %)	156 (7.5%)
7–8 years old (*n*, %)	61 (2.9%)
9–10 years old (*n*, %)	33 (1.6%)
Later (*n*, %)	100 (4.8%)
I am not transfusion dependent (*n*, %)	49 (2.4%)
Missing (*n*, %)	25 (1.2%)
What is your current transfusion regime?	
I am not transfused (*n*, %)	91 (4.4%)
I am regularly transfused (*n*, %)	1779 (85.9%)
I am occasionally transfused (*n*, %)	179 (8.6%)
Missing (*n*, %)	22 (1.1%)
If regularly transfused, what is the usual Hb level pre-transfusion?	
Less than 7 mg/dl (*n*, %)	492 (23.8%)
8–9 mg/dl (*n*, %)	970 (46.8%)
10–11 mg/dl (*n*, %)	339 (16.4%)
Over 11 mg/dl (*n*, %)	165 (8.0%)
Missing (*n*, %)	105 (5.1%)
Are blood supplies adequate at the centre you are transfused or are there delays in transfusion?	
No delays	877 (42.3%)
Occasional delays	973 (47.0%)
Delays are frequent so my Hb falls very low	172 (8.3%)
Missing (*n*, %)	49 (2.4%)
What kind of blood filtration is available at the clinic?	
Pre-storage	367 (17.7%)
Bedside	534 (25.8%)
None	253 (12.2%)
I don’t know	855 (41.3%)
Missing (*n*, %)	62 (3.0%)
At what age did you start receiving iron chelation therapy?	
1–4 years old (*n*, %)	885 (42.7%)
5–6 years old (*n*, %)	370 (17.9%)
7–8 years old (*n*, %)	196 (9.5%)
9–10 years old (*n*, %)	176 (8.5%)
Later (*n*, %)	359 (17.3%)
Missing (*n*, %)	85 (4.1%)
What chelation drugs do you use?	
Desferrioxamine (Desferal)	469 (22.6%)
Deferiprone (Ferriprox/L1)	274 (13.2%)
Deferasirox (Exjade)	694 (33.5%)
Combination	506 (24.4%)
Missing (*n*, %)	128 (6.2%)
How often do you receive chelation?	
I receive it regularly as prescribed	1470 (71.0%)
I do not receive it regularly	372 (18.0%)
I don’t receive iron chelation therapy	176 (8.5%)
Missing (*n*, %)	53 (2.6%)
How available are iron chelation drugs to you and at what dose?	
I always receive the chelation drugs in the quantity that I need them (at the right dose, continuous availability)	1243 (60.0%)
I receive a lower dose of chelation drugs than prescribed because of there isn’t a large enough quantity (poor supplies)	377 (18.2%)
I receive chelation drugs but not all the time because of interruptions in supply	328 (15.8%)
Missing (*n*, %)	123 (5.9%)
How often is your ferritin level measured?	
Every month	161 (7.8%)
Every two months	110 (5.3%)
Every three months	646 (31.2%)
Every six months	819 (39.5%)
Every twelve months	194 (9.4%)
Never	86 (4.2%)
Missing (*n*, %)	55 (2.7%)
Your current ferritin level is	
<500 ng/ml	213 (10.3%)
501–1000 ng/ml	325 (15.7%)
1001–2000 ng/ml	446 (21.5%)
2001–4000 ng/ml	444 (21.4%)
>4001 ng/ml	362 (17.5%)
I do not know	236 (11.4%)
Missing (*n*, %)	45 (2.2%)
How often is cardiac iron measured using T2*?	
Twice a year	131 (6.3%)
Annually/every year	429 (20.7%)
Every 2 years	175 (8.5%)
Rarely	359 (17.3%)
Never	909 (43.9%)
Missing (*n*, %)	68 (3.3%)
What is your latest T2* level?	
Under 6 ms	345 (16.7%)
7–10 ms	357 (17.2%)
11–20 ms	166 (8.0%)
Over 20 ms	211 (10.2%)
Missing (*n*, %)	992 (47.9%)
How is your liver iron measured?	
Liver biopsy	67 (3.2%)
MRI	701 (33.8%)
Not measured at all	1195 (57.7%)
Missing (*n*, %)	108 (5.2%)
What is your latest liver iron concentration?	
Less than 7 mg/kg of dry weight	132 (6.4%)
8–15 mg/kg of dry weight	89 (4.3%)
Above 16 mg/kg of dry weight	49 (2.4%)
I am not sure	330 (15.9%)
I don’t know	1355 (65.4%)
Missing (*n*, %)	116 (5.6%)
If paying out of pocket, which services are you paying for? (Tick all that apply) *	
Transfusion	718
Chelation pumps	308
Chelation drugs	822
Lab tests	1064
MRI	525
Hospitalisation	513
Multidisciplinary care *	603
No answer (*n*, %)	377 (18.2%)
Who pays for your treatment? (Tick all that apply) *	
Myself/my family	1245
Health insurance (private): mine	194
Health insurance (private): my employer’s	97
State-provided free healthcare	563
State-provided, partly free	335
Other	317
No answer (*n*, %)	58 (2.8%)
What specialist(s) do you visit, in addition to your main treating doctor? (Tick all that apply) *	
Heart specialist	776
Endocrinologist	572
Diabetologist (If separate from endocrinologist)	225
Psychologist	142
Liver specialist	368
Nephrologist	182
No answer (*n*, %)	785 (37.9%)
Where are you transfused? (Tick all that apply) *	
Haematology ward	518
Children’s ward	181
Transfusion centres	1238
Other	219
None answer (*n*, %)	62 (3.0%)
Are you satisfied with the quality and type of services you are receiving?	
Very unsatisfied (*n*, %)	288 (13.9%)
Unsatisfied (*n*, %)	340 (16.4%)
Neutral (*n*, %)	412 (19.9%)
Satisfied (*n*, %)	783 (37.8%)
Very satisfied (*n*, %)	220 (10.6%)
Missing (*n*, %)	28 (1.4%)
How would you rate access to your treatment?	
Very difficult (*n*, %)	289 (14.0%)
Difficult (*n*, %)	471 (22.7%)
Neutral (*n*, %)	604 (29.2%)
Easy (*n*, %)	549 (26.5%)
Very easy (*n*, %)	121 (5.8%)
Missing (*n*, %)	37 (1.8%)
If your answer to the previous question was “Very difficult” or “Difficult” please indicate why:	
High cost of travel to treating centre	322 (15.5%)
High cost of treatment	348 (16.8%)
High cost of both travel to the treating centre and treatment	358 (17.3%)
Missing (*n*, %)	1043 (50.4%)
How many days per year do you lose from education or work because of having to attend treatment for thalassaemia?	
None	383 (18.5%)
1–5 days	296 (14.3%)
6–10 days	111 (5.4%)
11–15 days	387 (18.7%)
16 or more days	778 (37.6%)
Missing (*n*, %)	116 (5.6%)

* The study participants could select more than one response to these questions, and the percentages add up to more than 100%. Therefore, percentages are not shown.

**Table 4:** Cumulative survey findings from different countries as categorised by WHO regions.

Categorised by WHO Regions						
	African Region	Region of the Americas	Eastern Mediterranean Region	European Region	Southeast Asian Region	Western Pacific Region
Study population						
*n*	10	12	1111	208	679	23
Age (in years)						
Mean, (SD)	27.8 (11.9)	35.4 (13.8)	26.2 (11.2)	41.1 (11.6)	21.4 (11.0)	35.1 (11.6)
Missing (*n*, %)	0 (0%)	0 (0%)	14 (1.3%)	2 (1.0%)	237 (34.9%)	0 (0%)
Patient/Parent						
Patient (*n*, %)	9 (90%)	9 (75%)	871 (78.4%)	196 (94.2%)	348 (51.3%)	19 (82.6%)
Parent (*n*, %)	1 (10%)	3 (25%)	226 (20.3%)	11 (5.3%)	329 (48.5%)	4 (17.4%)
Missing (*n*, %)	0 (0%)	0 (0%)	14 (1.3%)	1 (0.5%)	2 (0.3%)	0 (0%)
What is your gender?						
Male (*n*, %)	4 (40%)	7 (58.3%)	537 (48.3%)	70 (33.7%)	244 (35.9%)	6 (26.1%)
Female (*n*, %)	6 (60%)	5 (41.7%)	560 (50.4%)	138 (66.3%)	349 (51.4%)	17 (73.9%)
Missing (*n*, %)	0 (0%)	0 (0%)	14 (1.3%)	0 (0%)	86 (12.7%)	0 (0%)
Which of the following categories best describes your employment status?						
Employed, working full-time (*n*, %)	3 (30%)	7 (58.3%)	229 (20.6%)	85 (40.9%)	148 (21.8%)	10 (43.5%)
Employed, working part-time (*n*, %)	1 (10%)	1 (8.3%)	106 (9.5%)	25 (12.0%)	52 (7.7%)	2 (8.7%)
Not employed, looking for work (*n*, %)	4 (40%)	1 (8.3%)	390 (35.1%)	24 (11.5%)	95 (14.0%)	8 (34.8%)
Not employed, not looking for work (*n*, %)	2 (20%)	2 (16.7%)	139 (12.5%)	14 (6.7%)	356 (52.4%)	1 (4.3%)
Retired (*n*, %)	0 (0%)	0 (0%)	23 (2.1%)	42 (20.2%)	3 (0.4%)	1 (4.3%)
Disabled, not able to work (*n*, %)	0 (0%)	1 (8.3%)	193 (17.4%)	15 (7.2%)	20 (2.9%)	1 (4.3%)
Missing (*n*, %)	0 (0%)	0 (0%)	31 (2.8%)	3 (1.4%)	5 (0.7%)	0 (0%)
Which of the following best describes your current relationship status?						
Married (*n*, %)	2 (20%)	5 (41.7%)	381 (34.3%)	97 (46.6%)	201 (29.6%)	11 (47.8%)
Widowed (*n*, %)	0 (0%)	0 (0%)	17 (1.5%)	2 (1.0%)	9 (1.3%)	0 (0%)
Divorced (*n*, %)	0 (0%)	0 (0%)	11 (1.0%)	8 (3.8%)	2 (0.3%)	1 (4.3%)
Separated (*n*, %)	0 (0%)	1 (8.3%)	9 (0.8%)	6 (2.9%)	8 (1.2%)	0 (0%)
Cohabiting (*n*, %)	6 (60%)	1 (8.3%)	343 (30.9%)	28 (13.5%)	285 (42.0%)	0 (0%)
Single, never married (*n*, %)	2 (20%)	5 (41.7%)	293 (26.4%)	61 (29.3%)	152 (22.4%)	11 (47.8%)
Prefer not to answer (*n*, %)	0 (0%)	0 (0%)	30 (2.7%)	4 (1.9%)	18 (2.7%)	0 (0%)
Missing (*n*, %)	0 (0%)	0 (0%)	27 (2.4%)	2 (1.0%)	4 (0.6%)	0 (0%)
What is the highest level of school you have completed or the highest degree you have received?						
Less than high school degree	0 (0%)	1 (8.3%)	326 (29.3%)	24 (11.5%)	117 (17.2%)	6 (26.1%)
High school degree or equivalent (e.g., GED)	3 (30%)	3 (25.0%)	246 (22.1%)	61 (29.3%)	131 (19.3%)	5 (21.7%)
Some college but no degree	1 (10%)	1 (8.3%)	90 (8.1%)	13 (6.3%)	52 (7.7%)	3 (13.0%)
Bachelor’s degree	2 (20%)	1 (8.3%)	309 (27.8%)	60 (28.8%)	114 (16.8%)	7 (30.4%)
Master’s degree	3 (30%)	6 (50.0%)	66 (5.9%)	36 (17.3%)	42 (6.2%)	2 (8.7%)
Doctoral degree	1 (10%)	0 (0%)	12 (1.1%)	3 (1.4%)	5 (0.7%)	0 (0%)
Trade school	0 (0%)	0 (0%)	22 (2.0%)	10 (4.8%)	3 (0.4%)	0 (0%)
Missing (*n*, %)	0 (0%)	0 (0%)	40 (3.6%)	1 (0.5%)	215 (31.7%)	0 (0%)
If paying out of pocket, which services are you paying for? (Tick all that apply)						
Transfusion	0	0	275	16	411	10
Chelation pumps	1	0	231	8	62	4
Chelation drugs	1	1	391	20	401	3
Lab tests	7	6	524	41	464	14
MRI	7	1	350	30	130	2
Hospitalization	0	1	379	9	107	9
Multidisciplinary care	5	3	409	55	121	5
No answer (*n*, %)	1 (10%)	4 (33.3%)	158 (14.2%)	93 (44.7%)	111 (16.3%)	3 (13.0%)
Who pays for your treatment? (Tick all that apply)						
Myself/my family	8	6	648	57	500	13
Health insurance (private): mine	2	0	150	14	25	1
Health insurance (private): my employer’s	1	2	64	6	22	2
State-provided free healthcare	4	5	272	127	141	7
State-provided, partly free	2	0	248	47	31	4
Other	0	1	85	6	223	2
No answer (*n*, %)	1 (10%)	0 (0%)	35 (3.2%)	4 (1.9%)	11 (1.6%)	0 (0%)
How would you rate access to your treatment?						
Very difficult (*n*, %)	1 (10%)	1 (8.3%)	236 (21.2%)	7 (3.4%)	40 (5.9%)	1 (4.3%)
Difficult (*n*, %)	6 (60%)	1 (8.3%)	283 (25.5%)	21 (10.1%)	151 (22.2%)	5 (21.7%)
Neutral (*n*, %)	0 (0%)	4 (33.3%)	421 (37.9%)	60 (28.8%)	103 (15.2%)	8 (34.8%)
Easy (*n*, %)	2 (20%)	5 (41.7%)	113 (10.2%)	79 (38.0%)	338 (49.8%)	7 (30.4%)
Very easy (*n*, %)	1 (10%)	1 (8.3%)	31 (2.8%)	41 (19.7%)	42 (6.2%)	2 (8.7%)
Missing (*n*, %)	0 (0%)	0 (0%)	27 (2.4%)	0 (0%)	5 (0.7%)	0 (0%)
If your answer to the previous question was “Very difficult” or “Difficult” please indicate why:						
High cost of travel to treating centre	0 (0%)	1 (8.3%)	173 (15.6%)	20 (9.6%)	120 (17.7%)	4 (17.4%)
High cost of treatment	1 (10%)	2 (16.7%)	284 (25.6%)	9 (4.3%)	46 (6.8%)	4 (17.4%)
High cost of both travel to the treating centre and treatment	5 (50%)	1 (8.3%)	252 (22.7%)	11 (5.3%)	81 (11.9%)	2 (8.7%)
Missing (*n*, %)	4 (40%)	8 (66.7%)	402 (36.2%)	168 (80.8%)	432 (63.6%)	13 (56.5%)
How many days per year do you lose from education or work because of having to attend treatment for thalassaemia?						
None	2 (20%)	4 (33.3%)	248 (13.6%)	61 (29.3%)	58 (8.5%)	4 (17.4%)
1–5 days	1 (10%)	0 (0%)	195 (8.5%)	21 (10.1%)	67 (9.9%)	8 (34.8%)
6–10 days	1 (10%)	1 (8.3%)	62 (4.5%)	12 (5.8%)	30 (4.4%)	2 (8.7%)
11–15 days	2 (20%)	0 (0%)	127 (29.9%)	20 (9.6%)	234 (34.5%)	2 (8.7%)
16 or more days	4 (40%)	7 (58.3%)	399 (41.2%)	88 (42.3%)	270 (39.8%)	3 (13.0%)
Missing (*n*, %)	0 (0%)	0 (0%)	80 (2.3%)	6 (2.9%)	20 (2.9%)	4 (17.4%)

## Data Availability

Data are available upon formal request.

## Supplementary Materials

The following supporting information can be downloaded at: https://www.mdpi.com/article/10.3390/medicina60040650/s1, Section S1: Country list of International Report; Section S2: Survey Questionnaire; Section S3: Country list and languages of questionnaire.
