# Supplementary material for: Addressing Thalassaemia Management from Patients’ Perspectives: An International Collaborative Assessment
Source: Medicina (Kaunas). 2024 Apr 18;60(4):650. doi: 10.3390/medicina60040650 (PMC11052322; doi:10.3390/medicina60040650)
Supplement: Supplementary file 1 [file medicina-60-00650-s001.zip › medicina-2955898-supplementary.pdf]

Supplementary Materials

Section S1

In Section S1 we present the 36 countries included in the international healthcare management report and categorized into the five different continents (Europe, Middle East, Africa, Asia, America).

Countries examined through international databases	
A/A	Country
Europe	
1.	Albania
2.	North Macedonia
Middle East	
3.	Bahrain
4.	Jordan
5.	Kuwait
6.	Saudi Arabia
7.	Lebanon
8.	Oman
9.	Palestine (West Bank and Gaza)
10.	United Arab Emirates
Africa	
11.	Algeria
12.	Egypt
13.	Mauritius
14.	Morocco
15.	Tunisia
Asia	
16.	Azerbaijan
17.	Bangladesh
18.	Cambodia
19.	China
20.	India
21.	Indonesia

22.	Iran
23.	Iraq
24.	Laos
25.	Malaysia
26.	Maldives
27.	Myanmar
28.	Nepal
29.	Pakistan
30.	Philippines
31.	Sri Lanka
32.	Thailand
33.	Vietnam
America	
34.	Argentina
35.	Brazil
36.	Trinidad &Tobago

Section S2

In Section S2 we provide the English version of the survey tool (questionnaire) used in the second phase of the International Collaborative Assessment Project (TIF's Global Survey).

Original Questionnaire Text in English
Thalassaemia Patient Questionnaire: Healthcare Coverage and Services for Thalassaemia Patients
Patients'/ Parents' views on the healthcare services they receive in their country
About the Questionnaire
Section 1 – Profile
This questionnaire should be answered by thalassaemia patients over 15 years old, or the parents of patients with thalassaemia under the age of 15.
Your answers will be used to support TIF's mission to lobby for safe and better quality treatment services for thalassaemia patients worldwide.
Note: All information is anonymous and will be kept confidential at all times.
1. Please provide the following information:

a. Age (in years):
b. Country
c. City
d. Email
e. Association
f. The centre you are treated in
g. Height (cm)
h. Weight (kg)

2. I am (e.g., a patient; a parent; other-please specify):
   a. Patient
   b. Parent

3. What is your gender?
   a. Male
   b. Female

4. Which of the following categories best describes your employment status?
   a. Employed, working full-time
   b. Employed, working part-time
   c. Not employed, looking for work
   d. Not employed, NOT looking for work
   e. Retired
   f. Disabled, not able to work

5. Which of the following best describes your current relationship status?
   a. Married
   b. Widowed
   c. Divorced
   d. Separated
   e. Cohabiting
   f. Single, never married
   g. Prefer not to answer

6. What is the highest level of school you have completed or the highest degree you have received?
   a. Less than high school degree
   b. High school degree or equivalent (e.g., GED)
   c. Some college but no degree
   d. Bachelor degree
   e. Master degree
   f. Doctoral degree
   g. Trade School

Section 2 – Medical Information

7. If you are a patient, what is your diagnosis? If you are a parent, what is the diagnosis of your child?
   a. Beta Thalassaemia major
   b. Beta Thalassaemia intermedia
   c. HbH disease
   d. Other (please specify)

8. At what age did you start transfusion therapy?
    a. 1-4 years old
    b. 4-6 years old
    c. 6-8 years old
    d. 8-10 years old
    e. Later
    f. I am not transfusion dependent

9. How is your current transfusion regime?
    a. I am not transfused
    b. I am regularly transfused
    c. I am occasionally transfused

10. If regularly transfused, what is the usual Hb level pre-transfusion?
    a. Less than 7mg/dl
    b. 7-8mg/dl
    c. 8-9mg/dl
    d. 9-10mg/dl
    e. 10-11mg/dl
    f. Over 11mg/dl

11. Where do you receive your treatment at?
    a. Hospital/Clinic
    b. City

Section 3 - Quality of Services Received

Please answer the following questions to give us a clear indication of the quality of treatment you are receiving.

12. Are blood supplies adequate at the centre you are transfused or are there delays in transfusion?
    a. No delays
    b. Occasional delays
    c. Delays are frequent so my Hb falls very low

13. What kind of blood filtration is available at the clinic?
    a. Pre-storage
    b. Bedside
    c. None
    d. I don't know

14. At what age did you start receiving iron chelation therapy?
    a. 1-4 years old
    b. 4-6 years old
    c. 6-8 years old
    d. 8-10 years old
    e. Later

15. What chelation drugs do you use?
    a. Desferrioxamine (Desferal)
    b. Deferiprone (Ferriprox / L1)

c. Deferasirox (Exjade)
d. Combination

16. How often do you receive chelation?
    a. I take it regularly as prescribed
    b. I do not take it regularly
    c. I don't receive iron chelation therapy

17. How available are iron chelation drugs to you and at what dose?
    a. I always receive the chelation drugs in the quantity that I need them (at the right dose, continuous availability)
    b. I receive a lower dose of chelation drugs than prescribed because of there isn't enough quantity (poor supplies)
    c. I receive chelation drugs but not all the time because of interruptions in supply

18. How often is your ferritin level measured?
    a. Every month
    b. Every two months
    c. Every three months
    d. Every six months
    e. Every twelve months
    f. Never

19. Your current ferritin level is:
    a. <500ng/ml
    b. 500-1000ng/ml
    c. 1000-2000ng/ml
    d. 2000-4000ng/ml
    e. >4000ng/ml
    f. I don't know

20. How often is cardiac iron measured by T2*?
    a. Twice a year
    b. Annually/ Every year
    c. Every 2 years
    d. Rarely
    e. Never

21. What is your latest T2* level?
    a. Under 6ms
    b. 6-10ms
    c. 10-20ms
    d. Over 20ms

22. How is your liver iron measured?
    a. Liver biopsy
    b. MRI
    c. Not measured at all

23. What is your latest Liver Iron Concentration?
    a. Less than 7 mg/kg of dry weight
    b. 7-15 mg/kg of dry weight
    c. Above 15 mg/kg of dry weight

d. I am not sure
e. I don't know

24. If paying out of pocket, which services are you paying for? (Tick all that apply)
    a. Transfusion
    b. Chelation pumps
    c. Chelation drugs
    d. Lab tests
    e. MRI
    f. Hospitalisation
    g. Multidisciplinary Care*

*Multidisciplinary team care is comprised of at least one patient and multiple health professionals from several different disciplines such as cardiologists, hepatologists, and endocrinologists. Health professionals who participate in a multidisciplinary team, care, collaborate and communicate together in order to address as many aspects of a patient's care as possible.

25. Who pays for your treatment? (Tick all that apply)
    a. Myself/ my family
    b. Health insurance (private): mine
    c. Health insurance (private): my employer's
    d. State-provided free healthcare
    e. State-provided, partly free
    f. Other

26. What specialist(s) do you visit, in addition to your main treating doctor?
    a. Heart specialist
    b. Endocrinologist
    c. Diabetologist (if separate from endocrinologist)
    d. Psychologist
    e. Liver specialist
    f. Nephrologist

27. Where are you transfused?
    a. Haematology ward
    b. Children's ward
    c. Transfusion centres
    d. Other

28. Are you satisfied with the quality and type of services you are receiving?
    a. Very unsatisfied
    b. Unsatisfied
    c. Neutral
    d. Satisfied
    e. Very satisfied

29. How would you rate access to your treatment?
    a. Very difficult
    b. Difficult
    c. Neutral
    d. Easy
    e. Very easy

30. If your answer to Question 29 was "Very difficult" or "Difficult" please indicate why:<br>a. High cost of travel to treating center<br>b. High cost of treatment<br>c. High cost of both travel to the treating centre and treatment
31. How many days per year do you lose from education or work because of having to attend treatment for thalassaemia?<br>a. None<br>b. 1-5 days<br>c. 6-10 days<br>d. 11-15 days<br>e. 16 or more days
Thank you very much for your input!
Your answers matter a lot to the Thalassaemia International Federation (TIF) and will help us in developing and promoting services in your country to improve the quality of your treatment.
Online Survey Privacy Statement: This survey is published and managed by the Thalassaemia International Federation (TIF) in collaboration with the Open University of Cyprus. Please be aware that we take your concerns about privacy seriously and we make every reasonable effort to respect it.
We respect your trust and protect your privacy, and therefore will never sell or share this data with any third parties. The data is securely stored and only accessible by authorized personnel.

Section S3:

In Section S3 we describe the different languages to which the survey tool (anonymous questionnaire) was translated and distributed to the countries along with the method of distribution.

Languages of questionnaire distributed in different countries		
Language	Country	Survey Approach
Albanian	Albania	TIF approached 1 patient and 1 healthcare professional to disseminate the questionnaire.
Arabic	Egypt	TIF approached 1 caregiver and active member of a local patient organisation to act as a coordinator for the Eastern Mediterranean Region (EMR). The coordinator was trained and guided by TIF. Further to the coordinator, TIF also approached one local patient association for dissemination of the survey.

	Iraq	TIF approached 1 patient and 1 local patient association for dissemination.
	Jordan	TIF approached 1 patient and 1 local association for dissemination.
	Kuwait	TIF approached 2 patients for dissemination.
	Lebanon	TIF approached 1 patient, 1 reference centre and 1 healthcare professional for dissemination.
	Morocco	TIF approached 1 local association for dissemination.
	Palestine	TIF approached 1 local association and 1 healthcare professional for dissemination.
	Saudi Arabia	TIF approached 1 patient and 2 local patient associations for dissemination.
	Tunisia	TIF approached 1 patient and 1 local patient association for dissemination.
	United Arab Emirates	TIF approached 1 patient and 1 local patient association for dissemination.
Bengali	Bangladesh	TIF approached 1 patient and 2 local patient associations for dissemination.<br>The patient association collected 300 questionnaires.
Chinese	China	TIF approached 1 local patient association for dissemination.
English	ALL	TIF created a dedicated website, which hosted links to all survey languages, including English. Regular posts on social media were also made to create awareness of the survey. All points of contact received the survey in English and their local language, where applicable.
	Bulgaria	TIF approached 1 patient, 1 local patient association and 1 healthcare professional for dissemination.
	Romania	TIF approached 1 patient and 1 local patient for dissemination.
Farsi (Persian)	Iran	TIF approached 1 patient, 1 local patient association and 1 healthcare professional for dissemination.
French	France	TIF approached 2 patients, 1 local patient association and 1 healthcare professional for dissemination.

	Mauritius	TIF approached 1 local patient association and 1 healthcare professional for dissemination.
German	Germany	TIF approached 1 patient and 1 local patient association for dissemination. The patient was assigned as a coordinator for the country.
Greek	Greece	TIF approached 2 patients and 1 local patient association for dissemination.
	Cyprus	TIF approached 1 patient, 1 local patient association and 1 healthcare professional for dissemination.
Hindi	India	TIF approached 1 patient and 1 local patient association for dissemination.
Italian	Italy	TIF approached 1 patient, 2 local patient associations and 1 healthcare professional for dissemination.
Indonesian	Indonesia	TIF approached 1 local patient association for dissemination.
Slavic	North Macedonia	TIF approached 1 patient for dissemination.
Malay	Malaysia	TIF approached 1 patient, 1 local patient association and 1 healthcare professional for dissemination.
Nepali	Nepal	TIF approached 2 patients and 1 local patient association for dissemination.
Sinhala	Sri Lanka	TIF approached 1 patient and 1 local patient association for dissemination.
Tagalog & Visayas	Philippines	TIF approached 1 patient association for dissemination.
Turkish	Azerbaijan	TIF approached 2 patients and 1 local patient association for dissemination.
Urdu	Pakistan	TIF approached 1 patient and 1 local patient association for dissemination.
